# Comprehensive Genetic Analysis of Japanese Autosomal Dominant Sensorineural Hearing Loss Patients

**DOI:** 10.1371/journal.pone.0166781

**Published:** 2016-12-02

**Authors:** Yoh-ichiro Iwasa, Shin-ya Nishio, Shin-ichi Usami

**Affiliations:** Department of Otorhinolaryngology, Shinshu University School of Medicine, 3-1-1 Asahi, Matsumoto, Nagano, 390–8621, Japan; Ajou University, REPUBLIC OF KOREA

## Abstract

**Background:**

In general, autosomal dominant inherited hearing loss does not have a founder mutation, with the causative mutation different in each family. For this reason, there has been a strong need for efficient diagnosis methods for autosomal dominant sensorineural hearing loss (ADSNHL) patients. This study sought to verify the effectiveness of our analysis algorithm for the screening of ADSNHL patients as well as the usefulness of the massively parallel DNA sequencing (MPS).

**Subjects and Methods:**

Seventy-five Japanese ADSNHL patients from 53 ENT departments nationwide participated in this study. We conducted genetic analysis of 75 ADSNHL patients using the Invader assay, TaqMan genotyping assay and MPS-based genetic screening.

**Results:**

A total of 46 (61.3%) ADSNHL patients were found to have at least one candidate gene variant.

**Conclusion:**

We were able to achieve a high mutation detection rate through the combination of the Invader assay, TaqMan genotyping assay and MPS. MPS could be used to successfully identify mutations in rare deafness genes.

## Introduction

Hearing loss is one of the most frequent congenital disorders in infants, with one out of every 500 newborns having bilateral hearing loss[1]. It is reported that 50–60% of these cases show a genetic etiology, with 80% of those with a genetic etiology demonstrating autosomal recessive hearing loss, and 20% of them showing autosomal dominant hearing loss[2]. However, as over 80 genes have been reported to be associated with hearing loss an efficient genetic screening system is required for nonsyndromic hearing loss. We have been working to popularize genetic analysis in Japan and have revealed the genetic background of Japanese hearing loss patients. Invader screening for 13 genes/46 mutations is currently used in Japanese, and is able to identify the responsible mutations in approximately 30–40% of deafness patients[3, 4], accelerating the clinical application of genetic screening. However, most of mutations targeted by the Invader assay are autosomal recessive genes (*GJB2*, *SLC26A4*, *CDH23*, etc.). However, diagnosis has been possible for only a few patients with an autosomal dominant inheritance pattern. The main features of autosomal dominant sensorineural hearing loss (ADSNHL) are that 1) the severity and/or progression varies in each family and 2) only a small number of founder mutations have been identified and there is remarkable diversity in the mutations found in each family. Accordingly, there is a strong need for the efficient diagnosis for ADSNHL families.

Thirty-two deafness-causative genes have reported to be associated with ADSNHL (Hereditary Hearing loss Homepage; http://hereditaryhearingloss.org/). However, the exons of all of these genes are too numerous for analysis using Sanger sequencing.

Recently, targeted exon sequencing of selected genes using massively parallel DNA sequencing (MPS) technology has provided us with a potential tool with which to systematically tackle previously intractable monogenic disorders and improve molecular diagnosis[5]. We also have recently reported that target exon sequencing using MPS is a powerful tool for the identification of rare gene mutations in deafness patients[6–8].

In this study, we conducted the genetic analysis of 75 ADSNHL patients using the Invader assay, TaqMan genotyping assay and MPS-based genetic screening. While the invader assay and TaqMan genotyping assay can effectively detect the variants frequently found in Japanese patients based on the large data set of the Japanese hearing loss patients[4], MPS can further analyze a large number of genes comprehensively. The purpose of this study is to confirm the effectiveness of our analysis algorithm for the screening of ADSNHL patients as well as the usefulness of MPS.

## Subjects and Methods

### Subjects

Seventy-five Japanese ADSNHL patients from 53 ENT departments nationwide participated in this study. We considered that a family that had hearing loss pedigrees in two or more generations to demonstrate autosomal dominant inheritance. Written informed consent was obtained from all subjects (or from their next of kin, caretaker, or guardian on the behalf of minors/children) prior to enrollment in the project. This study was approved by the ethical committees of Shinshu University and each of the other participating institutions listed as follows (Sapporo Medical University, Akita University, Iwate Medical University, Tohoku University, Tohoku Rosai Hospital, Yamagata University, Fukushima Medical University, Jichi Medical University, Gunma University, Jyuntendo University, Yokohama City University, Tokai University, Mejiro University, National Rehabilitation Center, Nihon University School, Saitama Medical University, Tokyo Medical University, Jikei University, Abe ENT clinic, Toranomon Hospital, Kitasato University, Tokyo Medical Center Institute of Sensory Organs, International University Health and Welfare Mita Hospital, Jichi University Saitama Medical Center, Aichi Children’s Health Medical Center, Chubu Rosai Hospital, Mie Hospital, Kyoto University, Kyoto Prefectural University, Mie University, Shiga Medical Center for Children, Shiga Medical University, Osaka University, Kansai Medical University, Kobe University, Osaka Medical Center and Research Institute for Maternal and Children Health, Hyogo College of Medicine, Kobe City Medical Center General Hospital, Wakayama Medical University, Kouchi University, Hiroshima University, Hiroshima City Hiroshima Citizen Hospital, Yamaguchi University, Ehime University, Kyushu University, Fukuoka University, Kurume University, Nagasaki University, Kanda ENT Clinic, Miyazaki Medical College, Kagoshima University, Ryukyus University).

### Invader assay

We used the Invader assay for screening 46 known mutations in 13 known deafness genes (*GJB2*, *SLC26A4*, *COCH*, *KCNQ4*, *MYO7A*, *TECTA*, *CRYM*, *POU3F4*, *EYA1*, mitochondrial 12 s ribosomal RNA, mitochondrial tRNA(Leu), mitochondrial tRNA(Ser) and mitochondrial tRNA(Lys)). The detailed protocol was described elsewhere[4].

### TaqMan genotyping assay

TaqMan genotyping assay for 55 known mutations in six deafness genes (*SLC26A4*, *CDH23*, *KCNQ4*, *TECTA*, *OTOF*, and *WFS1*) was applied for all subjects using a custom TaqMan SNP Genotyping Assay (Applied Biosystems, Life Technologies), TaqMan genotyping master mix (Applied Biosystems, Life Technologies) and a Stepone Plus real-time PCR system (Applied Biosystems, Life Technologies) according to the manufacturer’s instructions. [9]

### MPS sequencing

#### Amplicon library preparation

Amplicon libraries were prepared using an Ion AmpliSeq™ Custom Panel (Applied Biosystems, Life Technologies) according to the manufacturer’s instructions for 63 genes reported to cause non-syndromic hearing loss. The detailed protocol was described elsewhere[10]. After preparation, the amplicon libraries were diluted to 20pM and equal amounts of 6 libraries for 6 patients were pooled for one sequence reaction.

#### Emulsion PCR and sequencing

Emulsion PCR and sequencing were performed according to the manufacturer’s instructions. The detailed protocol was described elsewhere[10]. MPS was performed with an Ion Torrent Personal Genome Machine (PGM) system using an Ion PGM™ 200 Sequencing Kit and an Ion 318™ Chip (Life Technologies).

#### Base call and data analysis

The sequence data were mapped against the human genome sequence (build GRCh37/hg19) with a Torrent Mapping Alignment Program. After sequence mapping, the DNA variant regions were piled up with Torrent Variant Caller plug-in software. After variant detection, their effects were analyzed using ANNOVAR software[11, 12]. The missense, nonsense, insertion/deletion and splicing variants were selected from among the identified variants. Variants were further selected as less than 1% of 1) the 1,000 genome database[13], 2) the 6,500 exome variants (http://evs.gs.washington.edu/EVS/), 3) the Human Genetic Variation Database (dataset for 1,208 Japanese exome variants)[14], 4) the 269 in-house Japanese normal hearing loss controls, and 5) 1000 control data in the deafness variation database[15].

To predict the pathogenicity of missense variants, we used 12 functional prediction software programs including ANNOVAR (SIFT, Polyphen2 HVID, Polyphen2 HVAR, LRT, Mutation Taster, Mutation Assessor, FATHMM, Radial SVM, LR, GERP++, PhyloP and SiPhy 29-way log odds).

#### Variant confirmation

All the variants found in this study were confirmed by Sanger sequencing using exon-specific custom primers, and segregation analysis was performed for the patients with pathogenic variants.

## Results

**Fig 1:**
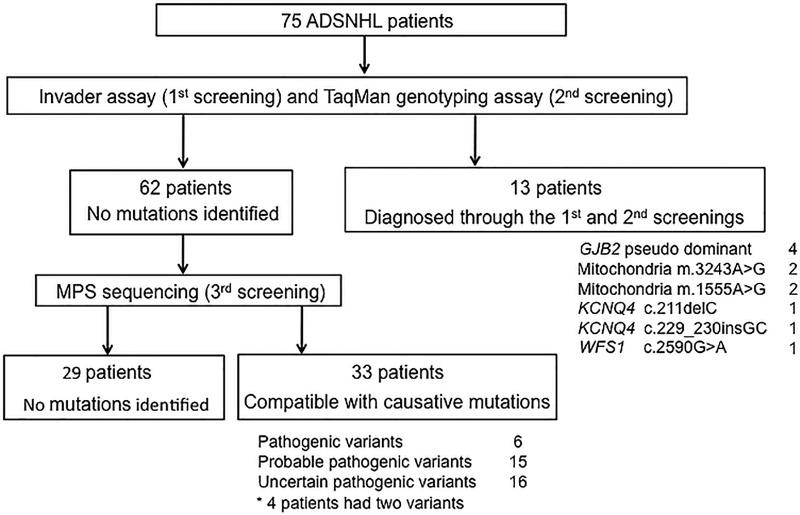
The overview of our analysis algorithm using 3-step genetic analysis (Invader assay, TaqMan genotyping assay and MPS).

A total of 46 (61.3%) of the 75 ADSNHL patients were found to have at least one candidate variant (Fig 1). Thirteen patients (17.3%; 13/75) were diagnosed through the 1^st^ and 2^nd^ screenings (Invader assay and TaqMan genotyping assay). Among the 62 patients who were not diagnosed through the 1^st^ and 2^nd^ screenings, 33 (44.0%; 33/75) were found to have some candidate variants through the 3^rd^ screening step (MPS).

### The 1^st^ screening (Invader assay)

The mutations found by the 1^st^ screening in this study are summarized in Table 1. Four patients (5.3%) had two *GJB2* mutations, and they were thought to belong to a pseudo-dominant family. Two patients (2.7%) had m.3243A>G mutations and 2 patients (2.7%) had m.1555A>G mutations. The 1^st^ screening was able to diagnose the responsible mutation in 8 (10.7%) of the 75 patients. Invader assay was thought to be useful for identifying pseudo-dominant and maternal inherited cases.

**Table 1:** The mutations found by the 1^st^ and 2^nd^ screenings in this study.

Patients	Gene	Nucleotide Change1	Nucleotide Change2	Amino acid Change1	Amino acid Change2
1^st^ screening					
1008	*GJB2*	c.235delC	c.[134G>A; 408C>A]	p.L79fs	p.[G45E; Y136X]
794	*GJB2*	c.235delC	c.[134G>A; 408C>A]	p.L79fs	p.[G45E; Y136X]
392	*GJB2*	c.427C>T	c.427C>T	p.R143W	p.R143W
1005	*GJB2*	c.109G>A	c.109G>A	p.V37I	p.V37I
505	Mitochondria	m.3243A>G	—	—	—
945	Mitochondria	m.3243A>G	—	—	—
508	Mitochondria	m.1555A>G	—	—	—
18	Mitochondria	m.1555A>G	—	—	—
2^nd^ screening					
982	*KCNQ4*	c.211delC	—	p.Q71fs	—
38	*KCNQ4*	c.211delC	—	p.Q71fs	—
780	*KCNQ4*	c.211delC	—	p.Q71fs	—
485	*KCNQ4*	c.229_230insGC	—	p.H77fs	—
416	*WFS1*	c.2590G>A	—	p.E864K	—

### The 2^nd^ screening (TaqMan genotyping assay)

The mutations found by the 2^nd^ screening in this study are summarized in Table 1. Four patients (5.3%) had *KCNQ4* mutations (c.211delC and c.229_230insGC), and one patient (1.3%) had a *WFS1* mutation (c.2590G>A, p.E864K). Thus, the 2^nd^ screening was able to diagnose the responsible mutation in 5 (6.7%) of the 75 patients. TaqMan genotyping assay identified *KCNQ4* mutations, which showed high GC contents.

### The 3^rd^ screening (MPS)

The 3^rd^ screening was performed for the 62 patients who were not diagnosed through the 1^st^ and 2^nd^ screenings. The mutations found by MPS in this study are summarized in Table 2. The identified mutations were classified into pathogenic variants, probable pathogenic variants and uncertain pathogenic variants. A classification of pathogenic variant was based on the following criteria; 1) previously reported as a pathogenic variant, 2) not identified in controls and 3) judged to be a damaging mutation by functional prediction software. Probable pathogenic variants were classified on the following criteria; 1) not identified in controls, 2) judged to be damaging mutations by functional prediction software and/or 3) nonsense, frameshift insertions/deletions or splicing junctions. Mutations identified in controls and/or not judged to be damaging mutations by functional prediction software were classified as uncertain pathogenic variants. We regarded a mutation as a pathogenic variant when more than six out of 8 prediction programs judged it to be a damaging mutation.

**Table 2:** The mutations found by the 3^rd^ screening in this study.

Patients	Gene		Nucleotide Change	Amino acid Change	Control (chromosome)	Functional Prediction								Severity*	Type	References
						SIFT	PolyPhen2	LRT	Mutation Taster	Mut Assesor	FATHMM	RadialSVM	LR			
Pathogenic variants																
964	*ACTG1*	NM_001614	c.353A>T	p.K118M	0/538	D(1)	B(0.40)	D(1)	A(1)	H(0.789)	D(0.54)	D(0/733)	D(0.932)	moderate	high	Zhu et al., 2003[30]
858	*COCH*	NM_004086	c.1115T>C	p.I372T	0/538	D(1)	D(0.996)	D(1)	D(1)	M(0.727)	D(0.456)	D(0.621)	D(0.746)	moderate	high	Tsukada et al., 2015[28]
962	*COCH*	NM_004086	c.1115T>C	p.I372T	0/538	D(1)	D(0.996)	D(1)	D(1)	M(0.727)	D(0.456)	D(0.621)	D(0.746)	severe	high	Tsukada et al., 2015[28]
883	*COL11A2*	NM_080681	c.3937_3948del12	p.1312_1315del4	0/538	NA	NA	NA	NA	NA	NA	NA	NA	mild	flat	Iwasa et al., 2015[32]
14	*MYO7A*	NM_000260	c.652G>A	p.D218N	0/538	D(0.99)	D(1)	D(1)	D(1)	M(0.736)	T(0.421)	D(0.558)	D0.61)	moderate	mid-high	Sun et al., 2011[29]
555	*WFS1*	NM_006005	c.2507A>C	p.K836T	0/538	T(0.38)	D(0.999)	D(1)	D(1)	L(0.566)	D(0.52)	DD(0.568)	D0.778)	mild	low	Fujikawa et al., 2010[27]
Probable pathogenic variants																
1051	*CCDC50*	NM_178335	c.820C>T	p.R274X	0/538	NA	NA	N(0.753)	A(1)	NA	NA	NA	NA	moderate	flat	This study
1043	*DIAPH1*	NM_005219	c.3637C>T	p.R1213X	0/538	T(0)	NA	D(1)	D(1)	NA	NA	NA	NA	severe	high	This study
610	*DIAPH1*	NM_005219	c.663G>C	p.L221F	0/538	D(1)	D(1)	D(1)	D(1)	M(0.679)	D(0.512)	D0.618)	D(0.827)	moderate	mid-high	This study
963	*EYA4*	NM_004100	c.1790delT	p.V597fs	0/538	NA	NA	NA	NA	NA	NA	NA	NA	moderate	flat	This study
954	*GRHL2*	NM_024915	c.937dupC	p.E312fs	0/538	NA	NA	NA	NA	NA	NA	NA	NA	moderate	flat	This study
946	*KCNQ4*	NM_004700	c.754G>C	p.A252P	0/538	D(1)	D(1)	D(1)	D(1)	M(0.75)	D(0.563)	D(0.523)	D(0.532)	moderate	mid-high	This study
995	*KCNQ4*	NM_004700	c.463G>A	p.G155R	0/538	D(1)	D(1)	D(1)	D(1)	L(0.60)	D(0.48)	T(0.30)	T(0.23)	moderate	high	This study
87	*MYH14*	NM_001077	c.823C>T	p.R275C	0/538	D(0.99)	D(1)	NA	D(1)	H(0.79)	T(0.423)	D(0.543)	D(0.595)	mild	mid	This study
1020	*MYO6*	NM_004999	c.897+2T>C	—	0/538	NA	NA	NA	D(1)	NA	NA	NA	NA	NA	NA	This study
1021	*MYO6*	NM_004999	c.1455T>A	p.N485K	0/538	D(1)	D(1)	N(1)	D(1)	H(0.877)	D(0.488)	D(0.587)	D(0.832)	moderate	flat	This study
433	*MYO6*	NM_004999	c.2287-2A>G	—	0/538	NA	NA	NA	D(1)	NA	NA	NA	NA	mild	mid	This study
694	*MYO7A*	NM_000260	c.479C>G	p.S160C	0/538	D(1)	D(1)	D(1)	D(1)	H(0.881)	D(0.545)	D(0.713)	D(0.97)	NA	NA	This study
673	*MYO7A*	NM_000260	c.1978G>A	p.G660R	0/538	D(1)	D(1)	D(1)	D(1)	H(0.882)	D(0.587)	D(0.696)	D(0.986)	mild	low	This study
1080	*TECTA*	NM_005422	c.4302C>A	p.Y1434X	0/538	T(0)	NA	D(1)	A(1)	NA	NA	NA	NA	moderate	low-mid	This study
963	*WFS1*	NM_006005	c.1147C>T	p.R383C	0/538	D(0.97)	B(0.039)	D(1)	D(1)	L(0.612)	D(0.492)	D(0.481)	D(0.588)	moderate	flat	This study
Uncertain pathogenic variants																
406	*COL11A2*	NM_080681	c.106C>T	p.R36W	0/538	D(1)	D(1)	N(0.999)	N(1)	L(0.619)	T(0.235)	T(0.264)	T)0.007)	moderate	mid-high	This study
981	*DIABLO*	NM_004403	c.92C>T	p.T31I	0/538	T(0.78)	D(1)	D(1)	D(1)	L(0.632)	T(0.436)	D(0.475)	D(0.585)	mild	flat	This study
962	*GRHL2*	NM_024915	c.1547G>A	p.R516Q	1/538	T(0.34)	B(0.037)	D(1)	D(1)	L(0.596)	T(0.293)	T(0.241)	T0.027)	severe	high	This study
970	*GRHL2*	NM_024915	c.1334A>G	p.Q445R	1/538	T(0.88)	B(0)	N(0.85)	N(0.711)	N(0.541)	T(0.294)	T(0.233)	T(0.022)	moderate	high	This study
97	*MYH14*	NM_001077	c.1990G>A	p.G664S	0/538	T(0.55)	P(0.796)	NA	D(0.976)	N(0.366)	T(0.421)	T(0.373)	T(0.228)	moderate	low-mid	This study
500	*MYH14*	NM_001077	c.1049G>A	p.R350Q	1/538	T(0.42)	B(0.136)	NA	D(0.793)	N(0.539)	D(0.479)	T(0.385)	T(0.388)	NA	NA	This study
962	*MYH14*	NM_001077	c.5324G>A	p.R1783H	0/538	D(1)	B(0.43)	NA	D(0.755)	M(0.708)	D(0.466)	D(0.534)	D(0.572)	severe	high	This study
433	*MYO6*	NM_004999	c.3497G>T	p.R1166L	1/538	T(0.89)	D(1)	D(1)	D(1)	L(0.633)	D(0.49)	D(0.584)	D(0.766)	mild	mid	This study
426	*MYO7A*	NM_000260	c.3701C>G	p.T1234S	1/538	D(1)	P(0.911)	D(1)	D(1)	M(0.683)	T(0.434)	D(0.482)	T(0.473)	moderate	high	This study
689	*MYO7A*	NM_000260	c.1868G>A	p.R623H	0/538	D(0.99)	D(1)	D(1)	D(1)	L(0.591)	T(0.42)	T(0.455)	T(0.44)	moderate	mid-high	This study
694	*MYO7A*	NM_000260	c.2947G>T	p.D983Y	0/538	T(0.94)	P(0.523)	D(1)	D(1)	M(0.698)	D(0.48)	D(0.592)	D(0.731)	NA	NA	This study
705	*MYO7A*	NM_000260	c.1436T>G	p.L479R	0/538	T(0.47)	B(0.292)	D(1)	D(1)	N(0.370)	T(0.413)	T(0.24)	T(0.088)	moderate	high	This study
502	*TECTA*	NM_005422	c.842T>C	p.V281A	0/538	T(0.15)	B(0.02)	N(0.989)	N(0.998)	L(0.577)	T(0.403)	T(0.254)	T(0.134)	moderate	high	This study
636	*TECTA*	NM_005422	c.5908G>A	p.A1970T	0/538	T(0.93)	B(0.056)	N(0.941)	N(0.999)	N(0.467)	D(0.457)	T(0.25)	T(0.262)	mild	mid-high	This study
853	*TJP2*	NM_004817	c.881G>A	p.S294N	0/538	NA	P(0.763)	D(1)	D(1)	M(0.681)	T(0.363)	T(0.275)	T(0.151)	moderate	mid	This study
541	*WFS1*	NM_006005	c.2359G>A	p.A787T	0/538	T(0.41)	B(0.003)	N(0.526)	N(1)	N(0.531)	D(0.514)	T(0.329)	T(0.485)	profound	flat	This study

*average 500, 1000, 2000 and 4000Hz in the better hearing ear. 25–39:mild, 40–69:moderate, 70–89:severe, 90-: profound

A, disease causing automatic; B, benign; C, conserved; D, deleterious or probably damaging or disease causing; H, high; L, low; M, medium; N, neutral or polymorphism; NA, not applicable; P, possibly damaging; T, tolerated

Based on the above criteria, six mutations were classified as pathogenic variants in 5 genes (*COL11A2*, *MYO7A*, *WFS1*, *ACTG1* and *COCH*), 15 were thought to be probable pathogenic variants in 11 genes (*CCDC50*, *DIAPH1*, *EYA4*, *GRHL2*, *KCNQ4*, *MTH14*, *MYO6*, *MYO7A*, *TECTA and WFS1*), and 16 were thought to be uncertain pathogenic variants in 8 genes (*COL11A2*, *DIABLO*, *GRHL2*, *MYH14*, *MYO6*, *MYO7A*, *TECTA*, *TJP2* and *WFS1*).

The 3^rd^ screening allowed at least one candidate mutation to be identified in 33 (44.0%; 33/75) of the 62 patients who were not diagnosed through the 1^st^ or 2^nd^ screenings.

## Discussion

The results of this study confirmed that our analysis algorithm is an efficient diagnostic strategy for ADSNHL patients. Overall, the 3-step genetic analysis enabled the detection of mutations in 46 (61.3%) of 75 families. Previous studies have reported diagnostic rates of 40.0% (4/10)[16] and 57.1% (4/7)[17] using only MPS. In this study, the number of patients analyzed was much higher than those in past reports, and we believe that this is the first report in which the study population contains a large number of ADSNHL patients.

In this study, we analyzed mutations in *GJB2* (for detecting pseudo-dominant cases), mitochondrial m.3243A>G, m.1555A>G (that were not analyzed by MPS) using the 1^st^ screening step. As a result, we detected 4 pseudo-dominant families (5.3%) resulting from *GJB2* mutations and 4 families (5.3%) with mitochondrial mutations. It is difficult to distinguish whether a family demonstrates dominant inheritance or not (pseudo-dominant and maternal inheritance) based only on family history, particularly in developed countries where families are usually small. It is, therefore, important to analyze autosomal recessive inheritance and maternal inheritance, even if the case appears to be autosomal dominant inheritance.

The 2^nd^ screening step allowed us to analyze mutations in six genes frequently detected in Japanese hearing loss patients. As a result, we detected causative mutations in 5 patients. *KCNQ4* mutations (c.211delC and c.229_230insGC) could not be detected by MPS because of technical limitations (extremely high GC contents), although this mutation was found frequently, as we previously reported[18]. We believe that the 2^nd^ screening step was particularly useful for detecting *KCNQ4* mutations.

The 3^rd^ screening step using MPS identified many rare mutations in a number of genes (*ACTG1*, *CCDC50*, *DIABLO*, *DIAPH1*, *EYA4*, *GRHL2*, and *TJP2*). It has been thought that mutations in many different genes are associated with each ADSNHL family; therefore, it was difficult to identify the causative mutation from the many candidates using only classical methods (Sanger sequencing, etc.). MPS is considered to be an effective tool for screening ADSNHL mutations. Further study is required to confirm the true causative mutations, however, newly identified rare mutations should provide a good detection marker for further use.

In the 3^rd^ screening step, 15 mutations were thought to be probable pathogenic variants. *CCDC50*: c.820C>T (p.R274X), *DIAPH1*: c.3637C>T (p.R1213X), *EYA4*: c.1790delT (p.V597fs), *GRHL2*: c.937dupC (p.E312fs), *MYO6*: c.2287-2A>G, *MYO6*: c.897+2T>C and *TECTA*: c.4302C>A (p.Y1434X) are truncating mutations; therefore, these mutations are speculated to show pathogenicity. In past reports, in particular, truncating mutations of *CCDC50*, *DIAPH1*, *EYA4*, *GRHL2* and *MYO6* genes were found to cause ADSNHL[19–24]. *DIAPH1*: c.663G>C (p.L221F), *KCNQ4*: c.754G>C (p.A252P), *MYH14*: c.823C>T (p.R275C), *MYO7A*: c.479C>G (p.S160C), *MYO6*: c.1455T>A (p.N485K), *MYO7A*: c.1978G>A (p.G660R) and *WFS1*: c.1147C>T (p.R383C) were judged to be damaging mutations by functional prediction programs and were not identified in controls. However, it was difficult to decide whether these mutations are the real cause of hearing loss as these mutations are missense mutations. Further study is needed to reach a definitive conclusion. *KCNQ4*: c.463G>A (p.G155R) did not meet the criteria for a probable pathogenic variant as only 5 out of the 8 prediction programs judged it to be pathogenic. However, the patient with this mutation showed ski slope hearing loss. In past reports, the patients with *KCNQ4* mutations showed a similar type of hearing loss. Their phenotype supports the pathogenicity of this mutation; therefore, we categorized this mutation as a probable pathogenic variant.

Two *KCNQ4* mutations were found through the 3^rd^ screening step. Therefore, a total of 6 (8.0%) of the 75 patients were thought to have *KCNQ4* mutations. *KCNQ4* mutations are considered to be the most important causative mutation in Japanese ADSNHL patients, as we reported previously[18]. *WFS1* and *COCH* gene mutations were also reported to be frequently identified causes of ADSNHL[25, 26]. In our study, 3 patients (4.0%) were identified with *WFS1* mutations and 2 patients (2.7%) with *COCH* mutations. These frequencies are relatively high and are compatible with those from past reports. It is worthy of note that these mutations (*WFS1*: c.2507A>C (p.K836T), *COCH*: c.1115T>C (p.I372T)) are recurrent mutations in Japanese patients[27, 28]. Therefore, they may be related to a founder effect. *MYO7A* (4.0%), *MYO6* (4.0%), and *DIAPH1* (2.7%) were also detected frequently.

Some mutations (*WFS1*: c.2590G>A (p.E864K), *MYO7A*: c.652G>A (p.D218N) and *ACTG1*: c.353A>T (p.K118M)) were previously reported in different populations[25, 29–31]. Therefore, they may be hot spot mutations rather than founder mutations. Until now, it has been thought that ADSNHL has a different causative mutation in each family and that it is difficult to diagnose ADSNHL families efficiently. However, even in cases of ADSNHL, hot spot mutations and founder mutations are considered to contribute to the etiology to some extent.

In conclusion, MPS was able to successfully identify mutations in rare deafness genes. Moreover, we achieved a high mutation detection rate through a combination of the Invader assay, TaqMan genotyping assay and MPS. The use of MPS is expected to provide a much fuller understanding of the genetic background in cases of ADSNHL.

## Abbreviations

ADSNHL: autosomal dominant sensorineural hearing loss
MPS: massively parallel DNA sequencing
ENT: ear nose throat
SNP: single nucleotide polymorphism
PCR: polymerase chain reaction
